# Evidence-Based Medical Management and Physiotherapy Rehabilitation in Pediatric Traumatic Brain Injury: A Narrative Review

**DOI:** 10.7759/cureus.69573

**Published:** 2024-09-17

**Authors:** Bhumala P Vaidya, H V Sharath, Neha A Brahmane, Raghumahanti Raghuveer, Moh'd Irshad Qureshi

**Affiliations:** 1 Department of Neuro-Physiotherapy, Ravi Nair Physiotherapy College, Datta Meghe Institute of Higher Education & Research (Deemed to be University), Wardha, IND; 2 Department of Paediatric Physiotherapy, Ravi Nair Physiotherapy College, Datta Meghe Institute of Higher Education & Research (Deemed to be University), Wardha, IND

**Keywords:** children, medical treatment, narrative review, physical rehabilitation, traumatic brain injury

## Abstract

Children between the ages of one and 18 are at a heightened risk of death and impairment due to traumatic brain injury (TBI). TBI can be deadly and is usually categorized as mild, moderate, or severe according to the Glasgow Coma Scale (GCS). For individuals with a TBI and an abnormal GCS, the preferred modality is non-contrast CT of the head. This review focuses on the medical treatment and rehabilitation of children with TBI and their outcomes. This was searched in databases such as PubMed, Google Scholar, and EMBASE. The literature search criteria included "traumatic brain injury AND physiotherapy rehabilitation OR medical treatment," with additional filters applied, including full text, both male and female, ages below 18 years, and publications from 2012 to 2024. This study conducted research on the treatment and rehabilitation of children with TBI. Ten randomized clinical trials, non-randomized trials, and longitudinal cohort study randomized trials met the inclusion criteria, which include hyperbaric oxygen therapy, melatonin, nabiximols, psycho-educational intervention, online therapy, the virtual reality rehabilitation program, and conventional occupational therapy. The results show significant improvement in forearm supination, performance in daily living, quality of life, reduced anger, improved school functioning, improvement in TBI conditions, reduced hospital stay, activity facilitating recovery, and reduced prolonged symptoms. This review has addressed the effectiveness of various medical management and rehabilitation strategies, which are important in TBI and aim to mitigate post-traumatic/concussion impact and improve quality of life. Articles regarding children's TBI rehabilitation are comparatively few. One possible solution to this issue would be to encourage further rehabilitation intervention trials.

## Introduction and background

Children between the ages of one and 18 are at a heightened risk of death and impairment due to traumatic brain injury (TBI) [1]. A head injury from a mechanical impact causes a disruption in normal brain activity, which is the hallmark of the illness [2]. According to the Glasgow Coma Scale (GCS), TBI is typically classified as mild, moderate, or severe. A TBI can be fatal. Individuals with a GCS score between 9 and 13 are defined as having moderate TBI, whereas those with a score between 14 and 15 are considered to have a mild TBI (mTBI). GCS scores ranging from 3 to 8 indicate a serious TBI. Severe TBI puts children at high risk for both neurological morbidity and mortality [3]. For children below the age of four, the traumatic injury mortality rate is five per 100,000 per year. Children under the age of four have a greater death rate than those between the ages of five and 14 [4].

Excessive distortion of the brain parenchyma and the vascular system surrounding the skull and its normal attachment sites is the primary cause of TBI [5]. Due to its substantial vascularization, the scalp can result in fatal blood loss. In a baby, child, or toddler, hemorrhagic shock can result from even a little loss of blood volume and can happen even in the absence of obvious exterior bleeding [6]. For individuals with a TBI and an abnormal GCS, non-contrast computed tomography (CT) of the head is the preferred modality. When the clinical picture is still not clear following CT imaging, a subtle lesion needs to be recommended [7]. The limited sensitivity of rapid magnetic resonance imaging has been shown in small, retrospective comparisons with CT [8]. However, most of the studies did not consistently use sequences that are most sensitive for detecting blood products, such as susceptibility-weighted imaging and gradient recall echo (GRE) [9].

## Review

Methodology

Articles were searched in databases such as PubMed, Google Scholar, and EMBASE. The literature search criteria included "Traumatic brain injury AND physiotherapy rehabilitation OR medical treatment," with additional filters applied, including full text, both male and female, ages below 18 years, and publications from 2012 to 2024. With these terms, a standard Google search was also carried out. Following the full-text review process, further inclusion criteria included empirical research and randomized controlled trials (Table 1).

**Table 1:** Literature review matrix pABI: pediatric acquired brain injury; pGMT: metacognitive training pediatric adaptation; BRIEF: Questionnaire Behavior Rating Inventory of Executive Function; BRI: Behavioral Regulation Index; MI: Metacognition Index; EF: executive functions; HBOT: hyperbaric oxygen therapy; IRC: internet resource comparison; CAPS: counselor-assisted problem solving; CAFAS: Child and Adolescent Functional Assessment Scale; mTBI: mild traumatic brain injury; PedsQL: pediatric quality of life; PSCI: post-concussion symptom inventory-youth; PPCS: persistent post-concussive symptoms; cMVPA: continuous motor vehicle physical therapy; HBI: health and behavior inventory; ICU: intensive care unit; TF-CBT: trauma-focused cognitive behavior therapy

Author and Date of Publication	Participation	Study Setting	Outcome Measure	Conclusion
Anne E. Brandt et al., 2021 [10]	Children aged 10 to 17 years with pediatric acquired brain injuries	This is an evaluator-blinded, parallel-RCT with a previously published trial protocol, set at two pediatric hospitals in Norway, St. Olavs Hospital, Trondheim University Hospital, and Oslo University Hospital, Rikshospitalet.	Questionnaire Behavior Rating Inventory of Executive Function (BRIEF); Behavioral Regulation Index (BRI); and Metacognition Index (MI)	In pediatric acquired brain injury (pABI), metacognitive training in pediatric adaptation (pGMT) showed no added benefit over psychoeducation on parent-reported daily life EF at 6 months. However, both were well-tolerated and improved different EF aspects.
Advait Prakash et al., 2012 [11]	A total of 54 patients with head injuries with GCS<8	In this study, each patient had three hyperbaric oxygen therapy (HBOT) sessions.	Glasgow coma scale, hospital stay, social behavior, disability reduction	This study suggests that for children suffering from traumatic brain injury, incorporating hyperbaric oxygen therapy significantly enhanced their outcomes and quality of life while lowering the likelihood of complications.
Shari L. Wade et al., 2015 [12]	This study includes 12 to 17-year-old children who have traumatic brain injury and were admitted to the hospital within the preceding seven months.	This study randomized participants to either the Internet resource comparison (IRC) condition or the counselor-assisted problem-solving condition.	Child and Adolescent Functional Assessment Scale (CAFAS), Internet Resource Comparison (IRC)	This study concludes that early access to internet therapy following an accident may enhance a child's functioning in the long run, especially for families from lower socioeconomic backgrounds. It is advisable to consider using CAPS clinically in the first several months following an accident.
Rosemarie Scolaro Moser et al., 2015 [13]	A total of 13 teenage sportsmen suffered a concussion and were still experiencing symptoms.	This single-group, non-randomized treatment study. To account for any spontaneous recovery, test data from three different time	Immediate Post-Concussion Assessment and Cognitive Testing	This study suggests that following education, reassurance, and a week of recommended rest, a significant number of adolescents with persistent symptoms after a concussion demonstrated improvements in their symptoms and cognitive abilities.
Irene Reneud et al., 2016 [14]	Participants between 6 to 18 who had suffered a minor traumatic brain injury	The study is a multicenter prospective longitudinal cohort study with a single-blind randomized controlled trial. Participants will be randomly assigned to either the psycho-educational intervention group or the usual care control group.	Child and adolescent scale of participation, children's assessment of participation and enjoyment, Pediatric quality of life scale, fatigue scale, Health behavior inventory, Impact of event scale, Family assessment, device-general functioning scale	The finding of this research will help to identify children with mTBI who may benefit from a psycho-educational intervention and are at risk for long-term participation issues.
Nancy A. Carney et al., 2017 [15]	A total of 132 individuals under 19 were diagnosed with severe, moderate traumatic brain injury.	In this study, a randomized controlled trial using a blinded outcome assessment. Its design was two groups in parallel.	Cognitive scale, quality of life module of the pediatric, quality of life inventory, the pediatric overall performance category, pediatric cerebral performance category,	In this study, they conclude that the PedsQL's family impact module scores showed a significant connection with the primary outcome measure. Six months after their injuries, families with children who fared better reported improved functioning conditions.
Karen M, Barlow 2020 [16]	A total of 99 children aged 8 to 18 experienced persistent post-concussive symptoms following a traumatic brain injury.	In this study, a single-center, randomized, double-blind, placebo-controlled trial was conducted to compare 3 mg melatonin with a placebo.	Post-concussion symptom inventory-youth(PSCI), behavioral, cognitive, and sleep problems, and functional impairment	In this study, they conclude that melatonin administered at 4 weeks post-injury did not significantly improve post-concussion symptoms in children with persistent post-concussive symptoms compared with a placebo. Between 1 and 3 months post-injury, 78% of children with PPCS showed a significant recovery.
Gauvin-lepege Jerome et al., 2020 [17]	A total of 393 pediatric patients aged 8 to 17 years showed symptoms 4 weeks after the injury.	This study is a multicenter prospective quasi-experimental control group design. Conducted in a tertiary care pediatric trauma center and community health care providers.	Child and parent ported level of post-concussion symptoms over the follow-up period, effect of fatigue, cognition, attention, and mood	This study concluded that beyond regular care, active rehabilitation intervention has little effect on post-concussion symptoms. Nonetheless, it improves their quality of life and reduces anger.
Ja Young Choi et al., 2021 [18]	A total of 70 children aged 3 to 18 have acquired brain injuries, have had it for at least 12 months, or have cerebral palsy.	The international randomized, controlled, single-blind, multicenter trial was conducted in rehabilitation institutions in Korea and China.	The upper limb physician's rating scale, Melbourne assessment of unilateral upper limb function-2, the pediatric evaluation of disability inventory computer adaptive test	This study concluded that both the virtual reality group and the control group showed substantial increases over baseline after treatment; however, the virtual reality group showed larger gains in upper-limb functions, forearm supination by kinematic analysis, and performance of daily living tasks.
Charlie Fairhurst et al., 2020 [19]	A total of 72 participants aged from 8 to 18 with spasticity.	11 sites in the UK, two in Israel, and one in the Czech used in the study. A 2:1 randomization was used to assign 72 participants who received ether nabiximols	In this study, they use spasticity, sleep quality, pain, health-related quality of life, comfort, depression, and safety as outcome measures, using a numerical rating scale (NRS).	This study shows that pediatric patients often tolerate oromucosal nabiximols well. Three occurrences of hallucinations were noted, though, one of which included an attempt at suicide and auditory hallucinations. Oromucosal nabiximols vs placebo did not reduce stiffness associated with cerebral palsy or central nervous system injuries.
Weihong Yuan et al., 2017 [20]	Twenty-two children (age: 15.83±1.77 years, 10 F) with 4–16 weeks of persistent symptoms after mTBI	A randomized clinical trial of aerobic training and stretching comparison combined with case-control comparison.	Five Global Network Measures The Self-reported Post-Concussion Symptom Inventory	This study found that brain connectivity analysis could potentially be used as a biomarker to detect brain abnormalities in children with lingering symptoms after mild traumatic brain injury.
Andrée-Anne Ledoux et al., 2024 [21]	A total of 267 individuals with acute concussions lasting less than 48 hours, ages 10 to 17.99 included.	The randomized clinical trial conducted at three Canadian pediatric emergency departments from March 2017 to December 2019 was used in this multicenter cohort analysis.	The total score on all 20 questions of the HBI. The cognitive and somatic symptom scores were constructed using the cumulative scores of 11 and 9 questions.	Children and adolescents who received 259 minutes of continuous motor vehicle physical therapy (cMVPA) in the first week and 565 minutes in the second-week post-concussion showed reduced symptoms. Higher cMVPA volumes at two weeks were associated with a 48% lower risk of persistent symptoms.
John J. Leddy et al., 2019 [22]	Male and female adolescent athletes (age 13-18 years)	This multicenter prospective randomized clinical trial was conducted at university concussion centers	Treadmill testing	Prescribing individualized aerobic exercise below the symptom threshold in the first week after a concussion may accelerate recovery and lower the risk of delayed recovery in adolescents.
Tim Dalgleish et al., 2015 [23]	Children aged 3 to 8 years with a principal diagnosis of post-traumatic stress disorder.	This study is a two-arm pilot randomized controlled trial comparing TF-CBT with treatment as usual.	Diagnostic Infant and Preschool Assessment	This study will identify areas where the intervention can be enhanced or changed before it is tested on a larger group of people.
Ericka L. Fink et al., 2020 [24]	Fifty-eight children between the ages of 3–17 years with new traumatic or non-traumatic brain insult	Three tertiary care pediatric ICUs in the United States.	Timing, treatment type, and frequency of deferrals and safety events	accelerate the pace and type of interventions but not the overall amount of rehabilitation.

Key findings and reasoning

Table 2 outlines the key findings and reasoning.

**Table 2:** Key findings pGMT: pediatric Goal Management Training; pBHW: pediatric Brain Health Workshop; TBI: traumatic brain injury; HBOT: hyperbaric oxygen therapy; CAPS: counselor-assisted problem solving; MTBI: mild traumatic brain injury; PPCS: persistent post-concussive symptoms; PSCI: post-concussion symptom inventory-youth; IRC: internet resource comparison; VR: virtual reality; CNS: central nervous system; NRS: numerical rating scale; MVPA: moderate-to-vigorous physical activity; cMVPA: cumulative moderate-to-vigorous physical activity; TF-CBT: trauma-focused cognitive behavior therapy; PTSD: post-traumatic stress disorder; TF-CBT-YC: trauma-focused cognitive behavior therapy in young children; ICU: intensive care unit

Key Finding	Reasoning
Developmental factors, duration of training, age at intervention, self-awareness, and contextual factors may all influence the effectiveness of metacognitive interventions. Metacognitive intervention (pGMT) did not outperform psychoeducation (pBHW) in reducing daily life executive dysfunction. The study explored factors like age and training duration that might influence the effectiveness of metacognitive training.	The study identifies several factors that may influence the effectiveness of metacognitive interventions, including developmental factors, duration of training, and self-awareness. The young brain's plasticity and vulnerability may influence the effectiveness of metacognitive training. The study explored potential differences in efficacy based on age.
HBOT may be beneficial in acute TBI by improving oxygenation, reducing inflammation, and promoting neuronal survival. The Glasgow Coma Scale (GCS) is widely used to assess TBI severity, but other factors like age, associated injuries, and hypoxia also predict outcomes.	HBOT has shown promise in animal models and human studies for improving outcomes in acute and chronic TBI. Understanding TBI severity is crucial for predicting outcomes and guiding treatment.
CAPS was particularly effective in improving functioning outside the home, such as school and community participation. Online problem-solving therapy (FPST) resulted in long-term improvements in functioning among adolescents with TBI.	CAPS helped improve school and community skills, which are important for long-term success. The CAPS treatment incorporated features that enhance tele-health effectiveness, such as therapist involvement and intensity.
Many athletes showed statistically reliable improvement on one or more cognitive domain scores. The study sample included a high proportion of athletes with neurodevelopmental problems or prior concussions. Education, reassurance, reduced stress, and increased sleep might contribute to the improvements.	The presence of neurodevelopmental problems or prior concussions might influence recovery outcomes. The study suggests that education, reassurance, reduced stress, and increased sleep might play a role in recovery.
This study aims to investigate activities, participation outcomes, and the effectiveness of an early psychoeducational intervention for children and adolescents with MTBI. A nested design was chosen to efficiently investigate the intervention's effectiveness. The study focuses on MTBI in children and adolescents. The study's intervention is designed to prevent activity and participation problems and is based on evidence from the literature.	The nested design allows for a faster investigation of the intervention's effectiveness while leveraging a large cohort. Focusing on MTBI allows a more targeted investigation of activities and participation outcomes. The intervention's specific theoretical basis and evidence-based design make it a valuable contribution to the field.
The study found a positive association between family function and functional outcomes in children with severe TBI. It also highlighted limitations in pre-hospital transport data and its impact on mortality calculations. Based on epidemiological data, the study found a lower incidence of severe pediatric TBI than expected.	The crucial role of family function in the recovery of children with severe TBI. Limitations in pre-hospital transport data make it difficult to accurately calculate mortality rates and generalize findings. The lower incidence of severe pediatric TBI than expected might be due to information gaps in the pre-hospital setting.
Melatonin did not significantly improve postconcussion symptoms, cognitive function, or quality of life in children with PPCS. Melatonin was associated with a decrease in externalizing problems. The study was randomized, placebo-controlled, and had a relatively large sample size.	The lack of a significant effect of melatonin might be due to factors like timing of treatment, duration of treatment, or individual differences in response. The observed decrease in externalizing problems suggests potential benefits for some children. The randomized placebo-controlled design and relatively large sample size make the study a valuable contribution to the field.
While PCSI scores were similar in both groups, patients in the ARI group reported a lower burden of symptoms and a significantly higher quality of life. This suggests that ARI can improve overall well-being despite not directly affecting symptom scores. Participants in the ARI group reported lower levels of anger compared to the control group, indicating potential benefits for emotional regulation.	The ARI intervention likely addresses multiple aspects of recovery, including physical, cognitive, and emotional components. This comprehensive approach may lead to improved overall well-being even if specific symptom scores remain similar. Quality of life is a subjective measure that reflects a person's overall perception of their health and well-being. ARI may have a positive impact on factors such as sleep, energy levels, and mood, which contribute to a better quality of life
The study suggests that VR rehabilitation can effectively enhance motor learning processes through implicit learning, concrete tasks, and focused attention. VR games were found to be more engaging and motivating for children compared to traditional occupational therapy sessions, potentially leading to increased neuroplasticity. The VR system incorporated task-specific training, which is a key principle in rehabilitation and can lead to improvements in upper-limb function and participation in daily activities.	The VR system provides opportunities for implicit learning, which is a more suitable approach for children and can facilitate skill acquisition. VR games offer concrete and relevant tasks that can guide movement and improve motor learning. VR games can focus the learner's attention on the results of their movements, which is more effective than attending to the movement itself. The VR system incorporated task-specific training principles, which can lead to improvements in functional skills.
Nabiximols did not demonstrate a significant difference in reducing spasticity compared to placebo in children with cerebral palsy or static post-traumatic CNS injury. Both nabiximols and placebo were generally well-tolerated, with a similar side effect profile.	There is a limited evidence base for the use of cannabinoids to treat spasticity in children, and this study did not provide sufficient evidence to support their efficacy. The primary endpoint, caregiver-reported spasticity 0 to 10 NRS, is subjective and may not accurately reflect objective changes in muscle tone.
Adolescents with persistent symptoms after mTBI showed initial evidence of abnormalities in global network connectivity measures. The aerobic training group exhibited significant changes in structural connectivity associated with the intervention, reflecting more typical connectivity patterns.	The study findings support the association between structural connectivity abnormalities and persistent symptoms after mTBI. Aerobic training may positively influence the recovery process by improving brain function and promoting neuroplasticity, but it could also be detrimental.
Engaging in higher volumes of moderate-to-vigorous physical activity (MVPA) within the first week or two weeks post-concussion was associated with lower symptom burden. · The benefits of cMVPA appeared to plateau after certain thresholds were reached. Exceeding 259 minutes in the first week or 375 minutes in the first two weeks might not provide additional benefits or could even have negative effects.	Physical activity can help stimulate recovery and reduce symptoms after a concussion. The observed threshold effects suggest that there may be an optimal range of cMVPA for symptom reduction. Exceeding this range might not provide additional benefits or could even be detrimental.
Individualized, progressive sub-symptom threshold aerobic exercise prescribed within one week of concussion was associated with improved recovery in adolescents with concussion symptoms compared to a placebo-like stretching intervention. Early aerobic exercise may help prevent some adolescents from experiencing delayed recovery, reducing the burden of social and academic problems.	Early aerobic exercise may stimulate recovery and improve brain function after a concussion. By addressing underlying pathologies and improving overall well-being, early aerobic exercise may help prevent delayed recovery.
There is currently no evidence base for the efficacy of Trauma-Focused Cognitive Behavioral Therapy (TF-CBT) in young children with PTSD. The growing recognition of PTSD in young children highlights the need for a treatment model specifically designed for this age group.	The study addresses a significant gap in the literature by investigating the effectiveness of TF-CBT for a population with limited treatment options. If found to be effective, TF-CBT-YC could provide a valuable treatment option for young children with PTSD, leading to improved functional outcomes and quality of life.
Early, protocolized ICU-based rehabilitation therapies were feasible to deliver in pediatric neurocritical care patients. Despite more ICU-based sessions in the Early Protocolized group, the total number of therapy sessions during the hospital stay was similar between groups. The therapies were delivered with relatively good safety profiles.	The study demonstrated the feasibility of implementing early ICU-based rehabilitation programs, even in the context of complex pediatric neurocritical care patients. The similar number of overall therapy sessions suggests that early intervention may not necessarily require a significantly increased workload for therapists. The study confirmed the safety of early ICU-based rehabilitation.

Discussion

This article focuses on physiotherapy rehabilitation and medical management and its impact on TBI in children. This article discusses various methods, such as hyperbaric oxygen therapy (HBOT), melatonin, nabiximols, psychoeducational intervention, online therapy, virtual reality rehabilitation programs, and conventional occupational therapy. The studies by Wade et al. (2015), Moser et al. (2015), and Carney et al. (2016) collectively examined interventions for recovery in children and adolescents following TBIs. Wade et al. found that counselor-assisted problem-solving (CAPS) led to better functional outcomes, particularly in children with less-educated parents, suggesting early online therapy's potential. Moser et al. showed that a one-week rest period improved cognitive performance in teenagers with persistent post-concussion symptoms, although the small sample size limits broader conclusions [12]. Carney et al. observed no significant differences between intervention and standard care groups at six months, highlighting the importance of family dynamics in recovery but raising concerns about intervention efficacy. These studies underscore the need to consider individual, familial, and therapeutic factors in designing effective TBI interventions for youth.

The studies by Prakash et al. (2012), Barlow et al. (2020), and Fairhurst et al. (2020) evaluate various therapies for managing brain injuries, focusing on efficacy and safety. Prakash et al. found that HBOT significantly improved cognitive functions and induced positive brain changes in severe brain injury patients, enhancing quality of life [10]. In contrast, Barlow et al. found no significant improvement with melatonin for pediatric post-concussion symptoms, questioning its viability as a standalone treatment. Fairhurst et al. observed that oromucosal nabiximols provided no significant benefit for children with spasticity due to cerebral palsy or brain injury, although they were generally well tolerated. These studies underscore the mixed outcomes of novel therapies and the need for further research to identify effective, safe treatments for brain injuries.

The studies by Renaud et al. (2016), Gauvin-Lepage et al. (2020), and Ledoux et al. (2024) collectively explored the effects of various interventions on children and adolescents recovering from mTBI and concussions. Renaud et al. focused on the activities and participation levels up to six months post-mTBI, identifying those who might benefit from psycho-educational interventions to mitigate long-term participation issues. Gauvin-Lepage et al. examined an active rehabilitation intervention (ARI) for young individuals, finding that while both ARI and standard care reduced post-concussion symptoms (PCS) over time, ARI offered additional benefits in overall well-being and functional recovery. Ledoux et al. highlighted the importance of optimizing early physical activity post-concussion, noting that while it aids in reducing immediate symptoms, its long-term impact may be limited. Together, these studies emphasize the importance of tailored interventions and the careful management of physical activity to support both short-term recovery and long-term participation in pediatric concussion and mTBI patients.

The findings from the studies conducted by Choi et al. (2020) and Luzinat et al. (2018) shed light on innovative rehabilitation and support methods for children with brain injuries. Choi et al. discovered that virtual reality rehabilitation using wearable sensors significantly improves upper-limb function, especially in children with severe motor impairments. Luzinat et al. stressed the value of organized camp programs in providing support for children with acquired brain injuries (ABI) and their families, laying the groundwork for future support initiatives. Both studies indicate that virtual reality and camp programs can play a crucial role in enhancing the recovery and support for children with brain injuries.

## Conclusions

Children between the ages of one and 18 are at a heightened risk of death and impairment due to TBI. The illness is characterized by abnormal brain activity caused by a head injury resulting from a physical impact. This review has addressed the effectiveness of various medical management and rehabilitation strategies that aim to reduce post-traumatic/concussion impact, improve quality of life and performance of daily living, reduce anger, improve school functioning, and reduce hospital stays. Early, active rehabilitation facilitates recovery and reduces prolonged symptoms. There is a relative scarcity of articles focused on the rehabilitation of children with TBI. A potential approach to address this issue could involve promoting additional trials focused on rehabilitation interventions.

## References

[1] Araki T, Yokota H, Morita A (2017). Pediatric traumatic brain injury: characteristic features, diagnosis, and management. Neurol Med Chir (Tokyo).

[2] Kochanek PM, Tasker RC, Bell MJ (2019). Management of pediatric severe traumatic brain injury: 2019 consensus and guidelines-based algorithm for first and second tier therapies. Pediatr Crit Care Med.

[3] Krasny-Pacini A, Chevignard M, Lancien S, Escolano S, Laurent-Vannier A, De Agostini M, Meyer P (2017). Executive function after severe childhood traumatic brain injury - age-at-injury vulnerability periods: the TGE prospective longitudinal study. Ann Phys Rehabil Med.

[4] Buchignani B, Beani E, Pomeroy V (2019). Action observation training for rehabilitation in brain injuries: a systematic review and meta-analysis. BMC Neurol.

[5] Lui A, Kumar KK, Grant GA (2022). Management of severe traumatic brain injury in pediatric patients. Front Toxicol.

[6] Riemann L, Zweckberger K, Unterberg A, El Damaty A, Younsi A (2020). Injury causes and severity in pediatric traumatic brain injury patients admitted to the ward or intensive care unit: a Collaborative European Neurotrauma Effectiveness Research in Traumatic Brain Injury (CENTER-TBI) Study. Front Neurol.

[7] Agrawal S, Abecasis F, Jalloh I (2024). Neuromonitoring in children with traumatic brain injury. Neurocrit Care.

[8] Haarbauer-Krupa J, Pugh MJ, Prager EM, Harmon N, Wolfe J, Yaffe K (2021). Epidemiology of chronic effects of traumatic brain injury. J Neurotrauma.

[9] Lindberg DM, Stence NV, Grubenhoff JA (2019). Feasibility and accuracy of fast MRI versus CT for traumatic brain injury in young children. Pediatrics.

[10] Brandt AE, Finnanger TG, Hypher RE (2021). Rehabilitation of executive function in chronic paediatric brain injury: a randomized controlled trial. BMC Med.

[11] Prakash A, Parelkar SV, Oak SN, Gupta RK, Sanghvi BV, Bachani M, Patil R (2012). Role of hyperbaric oxygen therapy in severe head injury in children. J Pediatr Neurosci.

[12] Wade SL, Kurowski BG, Kirkwood MW (2015). Online problem-solving therapy after traumatic brain injury: a randomized controlled trial. Pediatrics.

[13] Moser RS, Schatz P, Glenn M, Kollias KE, Iverson GL (2015). Examining prescribed rest as treatment for adolescents who are slow to recover from concussion. Brain Inj.

[14] Renaud MI, Lambregts SA, de Kloet AJ, Catsman-Berrevoets CE, van de Port IG, van Heugten CM (2016). Activities and participation of children and adolescents after mild traumatic brain injury and the effectiveness of an early intervention (Brains Ahead!): study protocol for a cohort study with a nested randomised controlled trial. Trials.

[15] Carney NA, Petroni GJ, Luján SB (2016). Postdischarge care of pediatric traumatic brain injury in Argentina: a multicenter randomized controlled trial. Pediatr Crit Care Med.

[16] Grima NA, Rajaratnam SM, Mansfield D, Sletten TL, Spitz G, Ponsford JL (2018). Efficacy of melatonin for sleep disturbance following traumatic brain injury: a randomized controlled trial. BMC Med.

[17] Gauvin-Lepage J, Friedman D, Grilli L, Sufrategui M, De Matteo C, Iverson GL, Gagnon I (2020). Effectiveness of an exercise-based active rehabilitation intervention for youth who are slow to recover after concussion. Clin J Sport Med.

[18] Choi JY, Yi SH, Ao L (2021). Virtual reality rehabilitation in children with brain injury: a randomized controlled trial. Dev Med Child Neurol.

[19] Fairhurst C, Kumar R, Checketts D, Tayo B, Turner S (2020). Efficacy and safety of nabiximols cannabinoid medicine for paediatric spasticity in cerebral palsy or traumatic brain injury: a randomized controlled trial. Dev Med Child Neurol.

[20] Yuan W, Wade SL, Quatman-Yates C, Hugentobler JA, Gubanich PJ, Kurowski BG (2017). Structural connectivity related to persistent symptoms after mild TBI in adolescents and response to aerobic training: preliminary investigation. J Head Trauma Rehabil.

[21] Ledoux AA, Sicard V, Bijelic V (2024). Optimal volume of moderate-to-vigorous physical activity postconcussion in children and adolescents. JAMA Netw Open.

[22] Leddy JJ, Haider MN, Ellis MJ (2019). Early subthreshold aerobic exercise for sport-related concussion: a randomized clinical trial. JAMA Pediatr.

[23] Dalgleish T, Goodall B, Chadwick I (2015). Trauma-focused cognitive behaviour therapy versus treatment as usual for post traumatic stress disorder (PTSD) in young children aged 3 to 8 years: study protocol for a randomized controlled trial. Trials.

[24] Fink EL, Beers SR, Houtrow AJ (2019). Early protocolized versus usual care rehabilitation for pediatric neurocritical care patients: a randomized controlled trial. Pediatr Crit Care Med.

